# Thrombotic Thrombocytopenic Purpura Presentation in an Elderly Gentleman Following COVID Vaccine Circumstances

**DOI:** 10.7759/cureus.16619

**Published:** 2021-07-25

**Authors:** Karthik Chamarti, Khavar Dar, Anand Reddy, Anika Gundlapalli, Denise Mourning, Kelash Bajaj

**Affiliations:** 1 Department of Internal Medicine, Texas Tech University Health Sciences Center at the Permian Basin, Odessa, USA; 2 Department of Critical Care Medicine, Odessa Regional Medical Center, Odessa, USA; 3 Department of Nephrology, Permian Basin Kidney Center, Odessa, USA; 4 Department of Hematology and Oncology, Texas Tech University Health Sciences Center at the Permian Basin, Odessa, USA

**Keywords:** covid-19 vaccine, thrombotic thrombocytopenic purpura, plasmapheresis, post vaccination complications, thrombotic microangiopathy (tma)

## Abstract

Thrombotic thrombocytopenic purpura (TTP) is a rare blood disorder that results in the formation of thrombi in the small blood vessels throughout the body. The two primary forms of TTP are acquired and familial forms. The acquired form usually presents in late childhood or adulthood. Almost 95% of the cases are due to an autoantibody directed against ADAMTS13, and the remaining 5% are due to drugs like ticlopidine, quinine, cyclosporine, gemcitabine, bevacizumab, and certain recreational drugs like ecstasy and cocaine. The familial forms present in infancy or early childhood, but sometimes they can present later in life. Management for acquired forms includes therapeutic plasma exchange and immunosuppressive agents. While for the hereditary forms, the mainstay of treatment is plasma infusion.

We present a case of an 80-year-old male with a known medical history of hypertension, type II diabetes mellitus, hyperlipidemia, gout, iron deficiency anemia, and Pfizer-BioNTech COVID-19 (coronavirus disease-19) vaccine administered two weeks before presentation to the ER for evaluation of generalized weakness and malaise. Laboratory findings showed severe anemia with hemoglobin of 4.8 g/dl, platelet count of 48 x 10^3/mcL, elevated lactate dehydrogenase (LDH), decreased haptoglobin, and peripheral smear showing schistocytes. The serum creatinine, total bilirubin, and troponin were elevated. All these findings were raising concern for presumptive diagnosis of TTP, which was confirmed with ADAMTS13 levels less than 10%. TTP was temporarily resolved in 10 days with plasma exchange therapy and high-dose corticosteroids.

It is difficult at this time to differentiate vaccine‐induced TTP from coincidental TTP presenting soon after vaccination. Further studies would be needed to understand better if this relationship between vaccination and TTP was coincidental or causal.

## Introduction

Thrombotic thrombocytopenic purpura (TTP) is a thrombotic microangiopathy (TMA) caused by severely decreased activity of the von Willebrand factor (VWF)-cleaving protease ADAMTS13. The pathogenesis is due to the formation of small-vessel platelet-rich thrombi that cause thrombocytopenia, microangiopathic hemolytic anemia (MAHA), and sometimes organ damage [1]. TTP is a medical emergency, and if appropriate treatment is not initiated promptly, it is almost always fatal. However, with proper treatment, survival rates of up to 90% are possible [1]. TTP can be acquired due to an autoantibody inhibitor (accounts for 95% of cases) or hereditary due to inherited mutations in ADAMTS13. The incidence of acquired TTP is approximately three cases per one million adults per year, based on data from the Oklahoma TTP-hemolytic uremic syndrome (HUS) Registry [2]. TTP is more common in females and people of African descent. The median age for the diagnosis of acquired TTP is 41 years, with a wide range (9-78 years) [1]. Acquired TTP is very unusual in patients more than 80 years of age. Here, we present a case of an 80-year-old male with TTP following Pfizer-BioNTech coronavirus disease-19 (COVID-19) vaccine administration circumstance. To the best of our knowledge, this is the first case reported in the medical literature so far.

## Case presentation

An 80-year-old Hispanic male with known medical problems of hypertension, type II diabetes mellitus, hyperlipidemia, gout, and iron deficiency anemia (baseline hemoglobin [Hb] of 11.1 g/dl) presented to the ER with complaints of generalized weakness and malaise for the past two days. The patient endorsed that he took his second dose of Pfizer-BioNTech COVID-19 vaccine two weeks before his presentation to the ER. The patient denied any fever, nausea, vomiting, diarrhea, chest pain, dyspnea, recent travel, sick contacts, drug abuse, and pedal edema.

On examination, the patient was in no acute distress. The patient's vitals were blood pressure of 154/84, heart rate of 88, respiratory rate of 22, saturating 99% on room air with a temperature of 37 degrees Celsius. He was pale and icteric. Oral examination showed purpura spots in the buccal mucosa. No cervical lymphadenopathy was noted. The abdomen was soft, non-tender, not distended, and no hepatosplenomegaly was noted. Respiratory and cardiovascular system examinations were normal. No focal neurological deficits were noted, power was 5/5 in all four extremities. Bilateral lower extremities were positive for purpuric spots.

**Figure 1:**
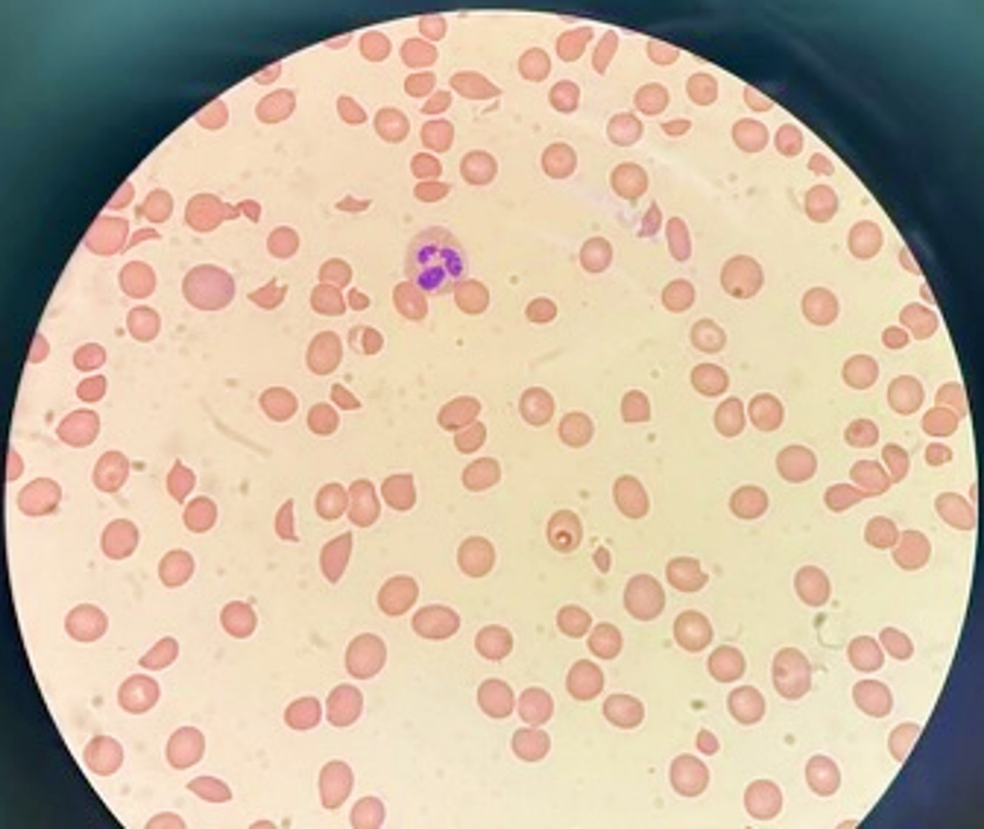
Peripheral blood smear demonstrating 3+ schistocytes.

Laboratory evaluation showed normocytic normochromic severe anemia with Hb of 4.8 g/dl, hematocrit was 14.2%, and thrombocytopenia with a platelet count of 48 x 10^3/mcL. His blood urea nitrogen was elevated at 75 mg/dl and creatinine of 2.4 mg/dL (baseline was 1 mg/dL). Liver function test showed total bilirubin of 3.3 mg/dL with direct bilirubin at 0.6 mg/dL and indirect bilirubin at 2.7 mg/dL. Prothrombin time was 12.2 and the international normalized ratio (INR) was 1.2. Lactate dehydrogenase (LDH) was elevated at 1118 U/L and haptoglobin was less than 10 mg/dL. Troponin was elevated at 3.810 ng/ml (Table 1). Peripheral blood smear showed 3+ schistocytes in each high-power field (Figure 1). All these findings raised concern for MAHA. The ADAMTS13 antibody inhibitor titer was 182%, and ADAMTS13 activity was less than 2%, which confirmed the diagnosis of TTP. Work up for anti-nuclear antibodies (ANA), anti-Smith antibodies, anti-double-stranded DNA (anti-dsDNA) antibodies, Scl-70 scleroderma antibodies, and anti-centromere antibodies were negative ruling out systemic lupus erythematosus, antiphospholipid syndrome, and systemic sclerosis as the cause for MAHA and thrombocytopenia. The stool culture was negative, and the patient did not have abdominal pain and diarrhea, ruling out Shiga toxin-mediated HUS as the cause for MAHA and thrombocytopenia. For the initial management of TTP, the patient received a transfusion with two units of packed RBCs and two units of platelets. The treatment was followed by prednisone 80 mg per oral daily and plasmapheresis. Over the subsequent 10 days, the patient recovered symptomatically, with laboratory data showing complete resolution of TTP. The patient was started on rituximab as an outpatient therapy to prevent relapse.

**Table 1:** Laboratory workup during the hospitalization course. Hb: Hemoglobin; Hct: Hematocrit; Plt: Platelet; BUN: Blood urea nitrogen; LDH: Lactate dehydrogenase; ADAMTS13: A disintegrin and metalloproteinase with a thrombospondin type 1 motif, member 13.

Laboratory test	Day 1 (Day of admission)	Day 2	Day 3	Day 4	Day 10 (Day of discharge)	Reference range
WBC	10.5	19.8	19	17.6	10.7	4–11 x10^3/mcL
Hb	4.8	7.2	7.3	6.7	8.5	14–18 g/dL
Hct	14.2	21.1	21.6	20.4	26.2	42%–50%
Plt	48	26	34	59	176	130 to 400 x 10^3/mcL
BUN	75	72	66	60	15	8–20 mg/dL
Creatinine	2.4	2.3	2.2	2.0	1.0	0.70–1.30 mg/dL
Total bilirubin	3.3	4.6	2.1	1.0	1.1	0.3–1.0 mg/dL
Indirect bilirubin	2.7	-	-	-	-	0.2–0.7 mg/dL
LDH	1118	-	397	-	-	80–225 U/L
Haptoglobin	<10	-	-	-	-	83–267 mg/dL
ADAMTS13	<2.0	-	-	-	-	>60%
Troponin	3.81	-	-	-	-	≤0.04 ng/mL

## Discussion

Acquired TTP is characterized by severe ADAMTS13 deficiency (typically, the activity levels are <10%) due to an autoantibody directed against ADAMTS13 [1]. ADAMTS13 is a plasma protease that functions by cleaving the ultra-large VWF produced by the endothelial cells and subsequently secreted into the plasma. This cleavage to smaller-sized multimers prevents ultra-large multimers from accumulating, thereby inhibiting the microthrombi formation that leads to thrombocytopenia and MAHA.

The clinical features of TTP include fever, neurological symptoms, MAHA, thrombocytopenia with bleeding or purpura, and renal dysfunction associated with hematuria or proteinuria or elevated blood urea nitrogen. These were referred to as typical pentad features of TTP. Due to advancements in our understanding of the pathogenesis and diagnosis of TTP, the percentage of patients with a typical pentad presentation has substantially decreased. Currently, the presence of thrombocytopenia and MAHA (increased LDH, reduced Hb and haptoglobin, and presence of schistocytes on peripheral smear) is sufficient for presumptive diagnosis of TTP. These features, along with the ADAMTS13 activity of less than 10% and increased ADAMTS13 antibody titer, confirm the diagnosis of TTP [3].

The treatment of choice for acquired TTP resulting from autoantibodies against ADAMTS13 is therapeutic plasma exchange (TPE) . With TPE, more than 60% of the TTP patients respond well [4]. Rock GA et al. in their randomized clinical trial demonstrated that plasma exchange was superior to plasma infusion for survival [5]. Other treatment strategies including corticosteroids, vincristine, cyclophosphamide, cyclosporine, rituximab (a humanized monoclonal antibody against B-cell surface antigen CD20), bortezomib (a proteasome inhibitor that targets plasma cells) [6, 7], and caplacizumab (a monoclonal antibody against VWF), may be prescribed in patients with acquired TTP to eliminate the formation of autoantibodies and maintain long-term remission. Since the pathogenesis of TTP involves the formation of platelet-rich thrombi in microcirculation, physicians treating patients with TTP are reluctant to use platelet transfusion. Swisher KK et al., based on their cohort, suggested that platelet transfusion should be minimized but can be considered in patients with overt bleeding and in patients undergoing invasive procedures with severe thrombocytopenia [8].

The Pfizer-BioNTech COVID-19 vaccine is an mRNA-type vaccine known as BNT162b2. Two vaccine doses are given 21 days apart. The most common side effects are pain, redness, swelling over the injection site, and systemic side effects: chills, fever, nausea, myalgia, and headaches [9]. Our patient who developed TTP following administration of Pfizer-BioNTech COVID-19 vaccine is the first case reported in the medical literature so far. Whether this relationship between vaccination and TTP is coincidental or causal remains the main question. There have been a total of nine reported cases of secondary immune thrombocytopenia (ITP) following the administration of the Pfizer vaccine so far [10]. The pathophysiology was due to autoantibody formation against platelet factor 4 in the absence of heparin exposure. Therefore, in our patient, we postulate there was autoantibody formation against the ADAMTS13.

The vaccine-associated autoimmunity is due to two main mechanisms: molecular mimicry and the rise of the cascade of immunological events leading to aberrant activation of the innate and acquired immune system. Molecular mimicry is attributed to either the cross-reactivity between antigens or the effect of adjuvant [11]. Aberrant activation of the immune system is due to the activation of several pro-inflammatory cascades like type 1 interferon and the nuclear translocation of the transcription factor nuclear factor-kappa B NF-kB [12]. When coming to the Pfizer-BioNTech COVID-19 vaccine (mRNA vaccine), this matter is further complicated by the nucleic acid formulation and the accelerated development process imposed by the emergency pandemic situation [13].

In summary, it is unclear whether the Pfizer-BioNTech COVID-19 vaccine can trigger de novo TTP. It is difficult at this time to differentiate vaccine‐induced TTP from coincidental TTP presenting soon after vaccination. To determine the true incidence of TTP post-vaccination, additional surveillance is required. If the Pfizer-BioNTech COVID-19 vaccine can cause TTP post-vaccination, we might anticipate many cases to be reported in the coming weeks to months as more people get vaccinated. It will be worthwhile to see whether exacerbations of other autoimmune conditions occur as well to gain a better perception of the host response to vaccination [10].

## Conclusions

Acquired TTP is a rare blood disorder characterized by the formation of thrombi in small blood vessels due to autoantibodies directed against ADAMTS13. Few research articles correlating the occurrence of COVID-19 vaccine-associated immune thrombosis and thrombocytopenia (VITT) have been published, but none with TTP. It is difficult at this time to differentiate vaccine‐induced TTP from coincidental TTP presenting soon after vaccination. Further surveillance safety data, along with experimental and clinical studies, are needed to assess the incidence of TTP post-vaccination.
